# Enhancing capacitive performance of magnetite-reduced graphene oxide nanocomposites through magnetic field-assisted ion migration

**DOI:** 10.1371/journal.pone.0292737

**Published:** 2024-02-07

**Authors:** Nur Alya Syakirah Abdul Jalil, Eslam Aboelazm, Cheng Seong Khe, Gomaa A. M. Ali, Kwok Feng Chong, Chin Wei Lai, Kok Yeow You

**Affiliations:** 1 Department of Fundamental and Applied Sciences, Universiti Teknologi PETRONAS, Seri Iskandar, Perak, Malaysia; 2 Center of Innovative Nanostructures and Nanodevices, Universiti Teknologi PETRONAS, Seri Iskandar, Perak, Malaysia; 3 Chemistry Department, Faculty of Science, Al-Azhar University, Assiut, Egypt; 4 Faculty of Industrial Sciences and Technology, Universiti Malaysia Pahang, Gambang, Kuantan, Malaysia; 5 Nanotechnology & Catalysis Research Centre, Institute of Advanced Studies, University of Malaya, Kuala Lumpur, Malaysia; 6 School of Electrical Engineering, Faculty of Engineering, Universiti Teknologi Malaysia, Skudai, Johor, Malaysia; Sungkyunkwan University, REPUBLIC OF KOREA

## Abstract

The transition towards renewable energy sources necessitates efficient energy storage systems to meet growing demands. Electrochemical capacitors, particularly electric double-layer capacitors (EDLCs), show promising performance due to their superior properties. However, the presence of resistance limits their performance. This study explores using an external magnetic field to mitigate ion transfer resistance and enhance capacitance in magnetite-reduced graphene oxide (M-rGO) nanocomposites. M-rGO nanocomposites with varying weight ratios of magnetite were synthesized and comprehensively characterized. Characterization highlighted the difference in certain parameters such as C/O ratio, the Id/Ig ratio, surface area and particle size that contribute towards alteration of M-rGO’s capacitive behaviour. Electrochemical studies demonstrated that applying a magnetic field increased specific capacitance by approximately 20% and reduced resistance by 33%. Notably, a maximum specific capacitance of 16.36 F/g (at a scan rate of 0.1 V/s) and 27.24 F/g (at a current density of 0.25 A/g) was achieved. These improvements were attributed to enhanced ion transportation and migration at the electrode/electrolyte interface, lowering overall resistance. However, it was also observed that the aforementioned parameters can also limit the M-rGO’s performance, resulting in saturated capacitive state despite a reduced resistance. The integration of magnetic fields enhances energy storage in nanocomposite systems, necessitating further investigation into underlying mechanisms and practical applications.

## 1. Introduction

The ever-growing demand for energy continues to rise daily, prompting many companies to explore renewable energy sources in order to keep pace with global requirements. The positive trend of increasing renewable energy generation in significant countries worldwide accentuates the need for energy storage to manage surplus energy effectively. Unlike non-renewable sources such as oil and gas, which can be physically stored and transported, the renewable energy sector faces a significant challenge in storing excess energy for future use. Since renewable sources often rely on the vagaries of nature, implementing energy storage solutions would be immensely beneficial in ensuring that energy demand can be met at any time of day.

Electrochemical capacitors (ECs) employ electric double layers (EDLs) rather than relying on redox reactions as their preferred energy storage mechanism. Due to their capacity to establish electric double layers via a highly reversible process and their inherent stability, ECs are extensively preferred as an energy storage method. EDLs are created by the nanometre-sized ion separation that occurs at the porous electrode’s interface and electrolyte when voltage is induced. This causes the opposing ions in the electrode and electrolyte to be attracted toward each other.

One of the promising materials currently employed in the advancement of energy storage is magnetite-reduced graphene oxide (M-rGO). Graphene, a two-dimensional material with an atomic thickness, is organized in a honeycomb crystal lattice structure. Various applications of graphene-based materials have been reported [1–5]. Reduced graphene oxide (rGO) is derived from the oxidation of graphite, aiming to replicate the advantageous properties of graphene, such as its electrical conductivity, while offering greater convenience in production compared to its parent material. The inclusion of magnetite (Fe_3_O_4_) further enhances the properties of rGO.

The growing interest in magnetite materials, including M-rGO is due to its potential to be controlled with an external magnetic field [6]. Magnetite, or Fe_3_O_4_, a superparamagnetic material, is favored due to its low degree of toxicity, chemically stable nature, and economical price point [7]. The formation of graphene-like material with a metal oxide can lead to improved super capacitive performance [8]. This poses the possibility of further enhancing the electrochemical properties of the capacitor by reducing the cell’s overall resistance with the assistance of a magnetic field of specific strength.

The overall impedance within the system comprises various components, including contributions from the electrolyte, electrode porosity, the contact interface between the electrode and the current collector, and other factors, as outlined in reference [9]. The primary source of resistance in an electrochemical capacitor originates from the interface between the electrode and electrolyte during the electric double layer formation, as well as at the junction between the electrode and the current collector. During the formation of the electric double layer, ion transfer resistance plays a critical role in determining the efficiency of electron transport and electrolyte ion penetration. Meanwhile, contact resistance affects the internal resistance within the carbon-based electrode material and the current collector. Ion transfer resistance contributes towards a large portion of the overall electrochemical cell resistance. The electron transportation and the electrolyte ion penetration influence ion transfer resistance. Low efficiency of electron transportation affects the cell’s efficiency during charging and discharging the cell while a low level of electrolyte ion penetration into inaccessible porous areas of the electrodes reduces the surface area of the created EDLs.

Another major contributor to the overall resistance within the cell originates from contact resistance. Contact resistance occurs between the interacting and contacting surfaces of the electrode and the current collector. Resistance within carbon-based electrodes can also be influenced by the presence and quantity of oxygen functional groups [10]. This directly influences the internal resistance contained within the electrode material. A higher concentration of specific oxygen functional groups can lead to a hindrance or reduction in the effective pore volume of the porous carbon electrode. This, in turn, affects the penetration of electrolyte ions and can impact the surface area of the electric double layers (EDLs) [11]. On the other hand, certain oxygen groups such as hydroxyl can improve the capacitors’ capacitance as it can further enhance the wettability of the electrode. Thus, instead of hindering ion migration, it can further encourage the phenomenon. Therefore, it can be summarized that the higher amount of certain oxygen functional groups on the surface of the carbon-based material in the electrode worsens the electrochemical performance of the overall cell.

This work aims to investigate the relationship between the magnetite content within the sample, and the effect of an external magnetic field on the electrochemical performance of the synthesized nanocomposites. In order to mitigate the aforementioned sources of resistance, the introduction of an external magnetic field is suggested as a means to enhance electrolyte penetration and facilitate transportation during the formation of the electric double layer. Further investigations can be conducted to gain a deeper understanding of how the attributes of M-rGO nanocomposites may additionally impact the electrochemical characteristics of the supercapacitor. It is evident that various factors can be interconnected and manipulated to further enhance these properties. There exists significant potential for innovation in supercapacitors through magnetic field manipulation, as the literature on this phenomenon remains relatively limited [12]. Furthermore, this study emphasizes the similar impact exhibited by the M-rGO nanocomposites in enhancing the electrochemical properties, thereby improving energy storage performance. By comprehending these two aspects, the influence of the magnetic field and the characteristics of the nanocomposites, a more comprehensive approach can be employed to significantly reduce the overall resistance encountered by the supercapacitor.

## 2. Experimental

### 2.1 Materials

The materials used in the preparation of magnetite-reduced graphene oxide (M-rGO) are graphene oxide prepared according to our previous work [6], deionized water, ammonium hydroxide (NH_4_OH), and iron (II) sulfate heptahydrate (FeSO_4_.7H_2_O).

### 2.2 Synthesis procedure

Graphene oxide (GO) was prepared as per previous work [6] where GO was synthesized with graphite power using the improved Hummers method. During the synthesis, 3.0 g of graphite powder was introduced to a mixture of 360 ml of sulphuric acid (H_2_SO_4_), 40 ml of phosphoric acid (H_3_PO_4_) and 18.0 potassium permanganate (KMnO_4_). This mixture was subjected to one hour of stirring while immersed in an ice batch. After the hour, the ice batch was discarded and continued to be stirred for 72 hours at 50°C in a water bath.

Afterwards, 400 ml of deionized water ice cubes and 8 ml of hydrogen peroxide (H_2_O_2_) were incorporated into the mixture until the colour transitioned to a bright yellow. Following this, the mixture was washed for 24 hours using 1M of hydrochloric acid (HCL), centrifuged again and washed with deionized wafer. The resulting GO samples were obtained and dried at 40°C.

Three different samples used different GO: FeSO_4_.7H_2_O weight ratios to vary the amount of magnetite (Fe_3_O_4_) within the nanocomposite. The three prepared ratios samples are coded as M-rGO18, M-rGO22, and M-rGO60 for being FeSO_4_.7H_2_O: GO of 1:18, 1:22, and 1:60, respectively. GO (0.2 g) was dispersed in 200 mL of deionized water and NH_4_OH (10 mL) is added to the solution until the solution has a pH of 11–12. On the other hand, the desired amount of FeSO_4_.7H_2_O was dissolved in 100 mL of deionized water. Once prepared, the GO solution was mixed with the prepared FeSO_4_.7H_2_O solution. The mixture was stirred at room temperature for a fixed duration of 6 h. The solution with precipitates was obtained at the end of the stirring period. This suspension was centrifuged and washed three times with deionized water, before drying at 400 degrees Celsius for up to 12 h or overnight. The resulting final product was the M-rGO nanocomposites.

### 2.3 Characterisations

The M-rGO nanocomposites were characterized using X-ray diffraction to obtain and evaluate the sample’s crystalline characteristics and to confirm the decoration of magnetite nanoparticles on the reduced graphene oxide sheets. X-ray diffraction measurements were carried out using Panalytical Xpert3 Powder model at a scan range of 5–95 degrees with a step size of 0.1 deg/step. The average crystallite size (D) was then obtained using the Scherrer equation (Eq 1) with the broadening of the peaks (β) obtained in the diffraction pattern.


$$D=\frac{K\lambda }{\beta cos\theta }$$ (1)

Where K represents the crystallite-shape factor, λ is the wavelength of the source of radiation, and θ is the Bragg angle. The morphology of the M-rGO nanocomposites were characterized with field emission scanning electron microscopy (FESEM) using the Tescan Clara model for imaging and energy dispersive spectroscopy (EDS). Images were collected at a magnification range of 10–200 kx. Using the images acquired at 200 kx, the particle size was calculated by obtaining the size of 100 particles and plotting a particle size distribution graph to evaluate the average size of the nanoparticles for each sample. In addition to the FESEM analysis, energy dispersive spectroscopy was performed to identify the elemental composition of iron, carbon, and oxygen within the nanocomposites. Fourier transform infrared spectroscopy (FTIR) was conducted to identify the functional groups within the nanocomposites using Perkin Elmer Frontier 01 model with a wavenumber range of 400–4000 cm^-1^. Besides that, Raman spectroscopy was performed and recorded using Horibba HR800 spectroscope to observe the peaks produced by the D-band and G-band within the nanocomposite. N_2_ adsorption-desorption analysis was done to obtain the sample’s surface area, pore volume, and pore diameter using Micromeritics Tristar 3020 model at a degassing temperature of 80°C and holding time of 240 minutes. In addition, vibrating sample magnetometry (VSM) measurements were taken using Lake Shore Cryotronics Lakeshore 340 model to measure the magnetic properties of the nanocomposites.

### 2.4 Electrochemical measurements

Evaluation of the electrochemical performance of the M-rGO nanocomposites by conducting cyclic voltammetry, galvanostatic charge-discharge, and electrochemical impedance spectroscopy. All tests were conducted with and without the magnetic field. The magnets were parallel towards the three-electrode set-up with a strength of approximately 1 Tesla. Before conducting the three electrochemical measurements, the working electrode is prepared using the M-rGO samples by combining 90% of the active material, 5% of carbon black, and 5% of polyvinylidene fluoride (PVDF). The slurry mixture is then coated onto the nickel foam. The counter electrode was a platinum wire, the reference electrode was Hg/HgO, and 1 M potassium hydroxide (KOH) as an electrolyte. Cyclic voltammetry was performed at a potential window of -0.65 V to 0.2 V (vs. Hg/HgO) at scan rates of 0.05, 0.1, and 0.5 V/s. Electrochemical impedance spectroscopy was conducted at a frequency of 100 kHz to 10 mHz with an alternating current dependent on the mass of active material on the nickel foam, while galvanostatic charge-discharge was performed at the same potential window at current densities of 0.25, 0.5, 1, 2, and 3 A/g.

## 3. Results and discussion

### 3.1 Characterisation of M-rGO nanocomposites

#### 3.1.1 Crystallite size and identification of successful M-rGO synthesis through characteristics peaks using X-ray diffraction

**Fig 1:**
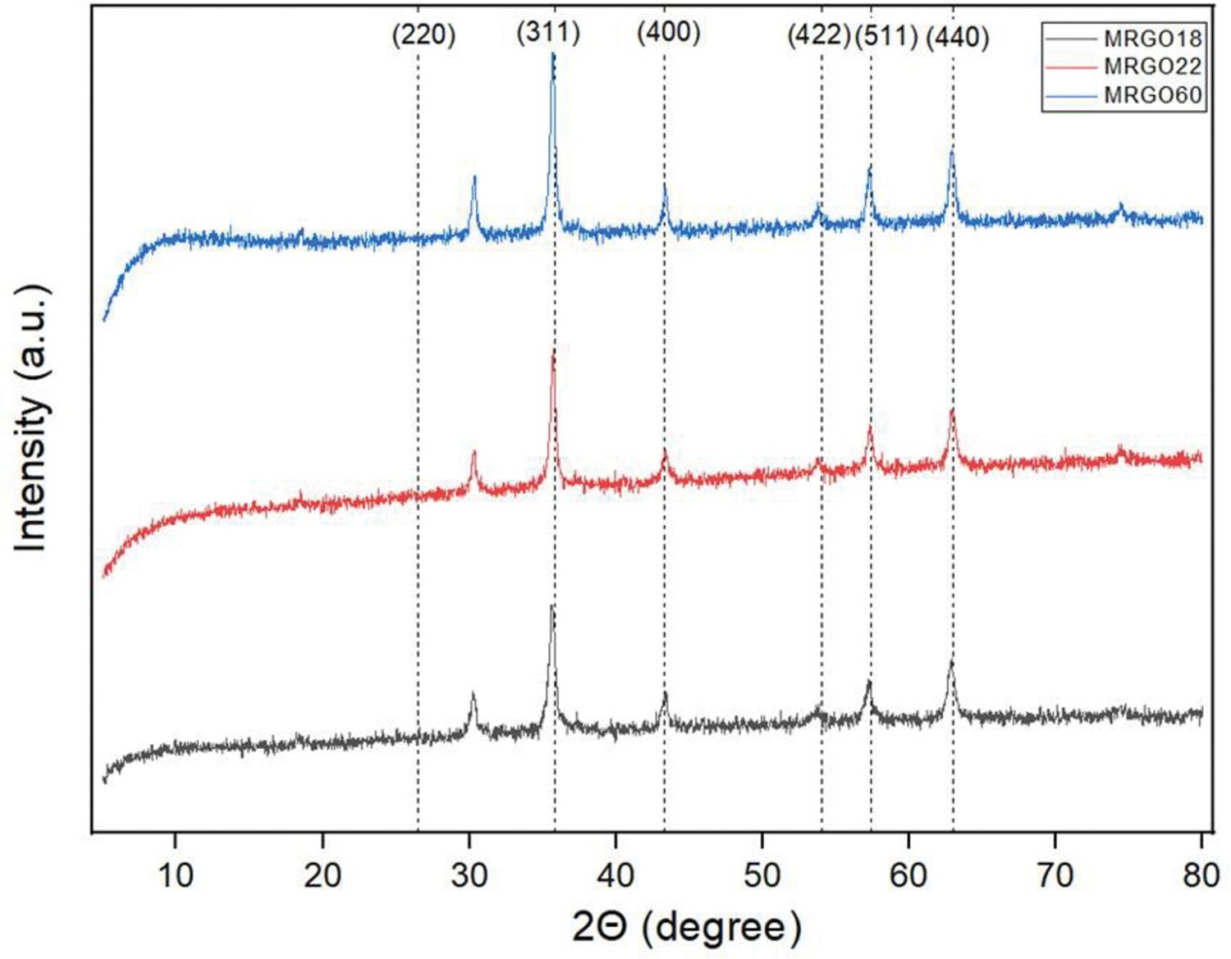
XRD patterns of M-rGO18, M-rGO22, and M-rGO60.

To further investigate, identify and confirm the synthesis of M-rGO nanocomposites, XRD was utilized to obtain the characteristic peaks of the sample. The synthesis process involved two separate processes: the reduction of GO into rGO and the deposition of magnetite (Fe_3_O_4_) on the rGO nanosheets. Referring to the XRD profiles in Fig 1, it can be summarized that M-rGO was successfully synthesized for all three ratio samples, M-rGO18, M-rGO22, and M-rGO60. The characteristic peak for GO in an XRD profile is usually observed at 2θ ≈ 10.40 at the crystal plane (001) [7]. Referring to Fig 1, no visible peak is detected within this region, which confirmed that GO was reduced to rGO. The absence of the GO peak within the peak profile highlights the complete reduction of GO due to adding the reducing agent of ammonium hydroxide [6]. On the other hand, the deposition of Fe_3_O_4_ can be identified by observing the 6 characteristic peaks of the magnetite. When no other peaks besides, this demonstrated successful deposition of magnetite onto the rGO sheets [6]. In addition to this, it is stated that the rGO peak was not visible due to the weaker crystallinity of the rGO compared to the magnetite material [6]. The absence of the rGO peak could be due to the small amount of rGO compared to magnetite within the nanocomposite, resulting in a lower diffraction intensity.

In correspondence with the ICDD no. 19–0629 for magnetite materials, the XRD peak profile obtained for all three samples of M-rGO18, M-rGO22, and M-rGO60 showed 6 diffraction peaks at 2θ≈ 30.3˚, 35.8˚, 43.3˚, 54.0˚, 57.4˚, and 63.0˚ as seen in Fig 1, corresponding to the crystal planes of (220), (311), (400), (422), (511) and (440), respectively [13, 14]. It is observed that the intensity of each sample on the respective diffraction peaks was similar with no obvious change in intensity despite the increase in the amount of magnetite. Thus, it can be concluded that all three M-rGO samples could be synthesized through in-situ chemical synthesis.

The crystallite size of the magnetite nanoparticles formed by the three weight ratios can be obtained using Eq (1). The average crystallite size obtained for M-rGO18, M-rGO22, and M-rGO60 was 15.86, 24.92, and 25.37 nm, respectively. The crystallite sizes varied distinctively and increased as the amount of magnetite in each sample of different weight ratios. This coincided with the FESEM images whereby the average particle size for each sample also increased. This can be further contributed to any possible agglomeration that occurs on the surface of rGO sheets during synthesis. Increasing the amount of magnetite within the sample can cause the rGO sheet to be packed, hence causing a possible tendency in agglomeration with its neighboring particles to form larger particles [6].

#### 3.1.2 Sample surface morphology, particle size and elemental composition of M-rGO nanocomposites using field emission scanning electron microscopy

**Fig 2:**
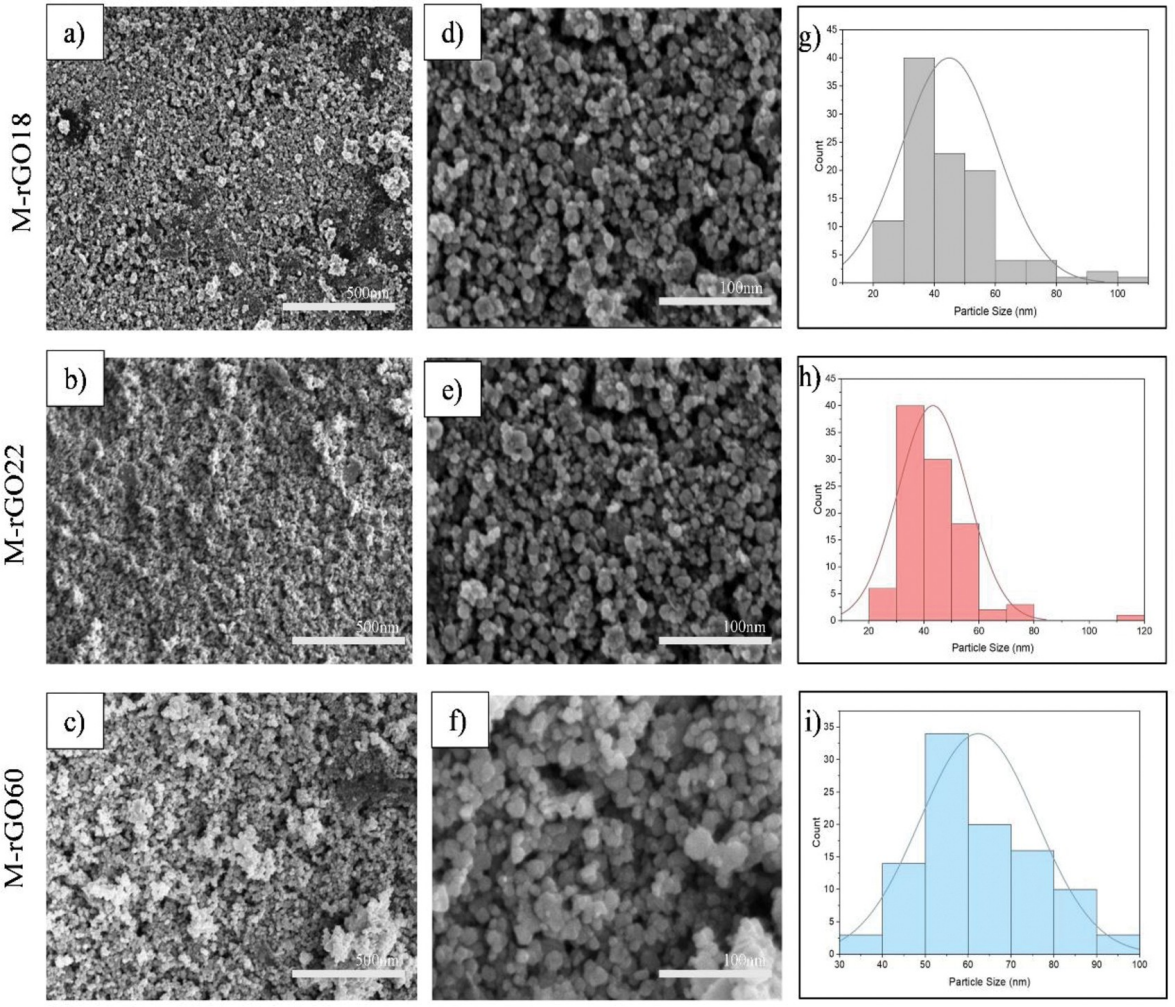
FESEM images at 50 kx (a, b, c), 200 kx (d, e, f), and the particle size distribution (g, h, i) for M-rGO18, M-rGO22, and M-rGO60.

With the confirmation of M-rGO synthesis from the XRD profile, FESEM was utilized for further study on the sample surface morphology and particle size of the nanocomposites obtained. Fig 2 demonstrates the FESEM images obtained for M-rGO18, M-rGO22, and M-rGO60 at 50 k magnification. The FESEM image obtained showed the rGO sheet acting as a platform for the deposition of the magnetite particles atop the nanosheet. The FESEM image in Fig 2 demonstrates how the magnetite nanoparticles were uniformly distributed across the rGO sheet at different densities for each weight ratio [7]. When comparing the three samples, it can be observed that the magnetic nanoparticles were more pronounced in covering a larger area of the rGO sheet for the M-rGO sample with a higher weight ratio of magnetite. For M-rGO60 (Fig 2C), the rGO sheet is more densely decorated with larger nanoparticles than the other two samples. In comparison, the rGO sheet is still visible in the background of M-rGO18 in Fig 2A due to its lower amount of magnetite.

The particle size for each sample can be observed in Fig 2. From the images, slight agglomeration of the magnetic nanoparticles can be seen in samples such as M-rGO22 and M-rGO60. As previously stated in the XRD analysis, the larger-sized nanoparticles may be due to the increased magnetite amount in the samples. Despite this, the rGO sheets and the magnetite particles were bonded covalently, thus the agglomeration was minimal as the rGO sheets were able to reduce the possibility of agglomeration [6]. Therefore, the size differences between the samples between M-rGO18 and M-rGO22 are not very disparate as the amount of magnetite in both samples merely varies by a small percentage. However, M-rGO60’s average size of particles is much larger in comparison as it has almost thrice the weight ratio of M-rGO22.

To obtain the average size of the nanoparticles, a total of 100 particles were selected at 200 kx. The average size of the nanoparticles of M-rGO18, M-rGO22, and M-rGO60 were 44.85 ± 15.58 nm, 46.63 ± 13.43 nm, and 62.35 ± 13.63 nm, respectively. Detected that increasing the amount of magnetite within the sample will form a more uniform size of nanoparticles. This is clearly shown by the distribution curves and histograms of each sample in Fig 2. M-rGO60’s formation of its nanoparticles had the most uniform distribution of size compared to the other two samples.

In addition to the FESEM analysis, EDS analysis was conducted to identify the composition of Fe, C, and O elements within the samples. This allowed for further investigation into the C/O within the nanocomposites. The C/O ratio heavily affects the functionality of these nanoparticles due to its role in altering the electrochemical properties of carbon-based materials such as their conductivity and pore size distribution, hence affecting their capacitive performance [10]. Essentially, an increase of certain oxygen functionalized groups within the carbon sample would further increase the overall contact resistance at the electrode/current collector interface leading towards a reduced conductivity [15]. Despite M-rGO60 having the highest concentration of oxygen within its sample, additional analysis such as FTIR and N_2_ adsorption-desorption indicate its higher conductivity due to the type and concentration of oxygen-functionalized groups that remain within the sample itself that plays the additional role. Further correlation between the types of oxygen-functionalized groups and their effect on the overall capacitive performance can be observed through FTIR and N_2_ adsorption-desorption analyses. In summary, certain oxygen-functionalized groups prevent further ion migration into the micropores, resulting in low capacitive performance. Graphene oxide with its more abundant amount of oxygen-functionalized groups hinders GO’s electrical conductivity properties [16]. Referring to Table 1, M-rGO60 has the lowest C/O ratio, followed by M-rGO18 and M-rGO22. From this ratio, it was predicted that M-rGO60 would also have the largest capacitance compared to the other samples.

**Table 1:** EDS analysis of Fe, C, and O in M-rGO18, M-rGO22, and M-rGO60.

Sample	C (Wt.%)	O (Wt.%)	Fe (Wt.%)	C/O ratio
M-rGO18	7.1	21.0	71.9	0.338
M-rGO22	12.7	24.5	62.8	0.518
M-rGO60	8.4	26.4	65.2	0.318

#### 3.1.3 Chemical bonding and structure of M-rGO nanocomposites using fourier transform infrared spectroscopy

**Fig 3:**
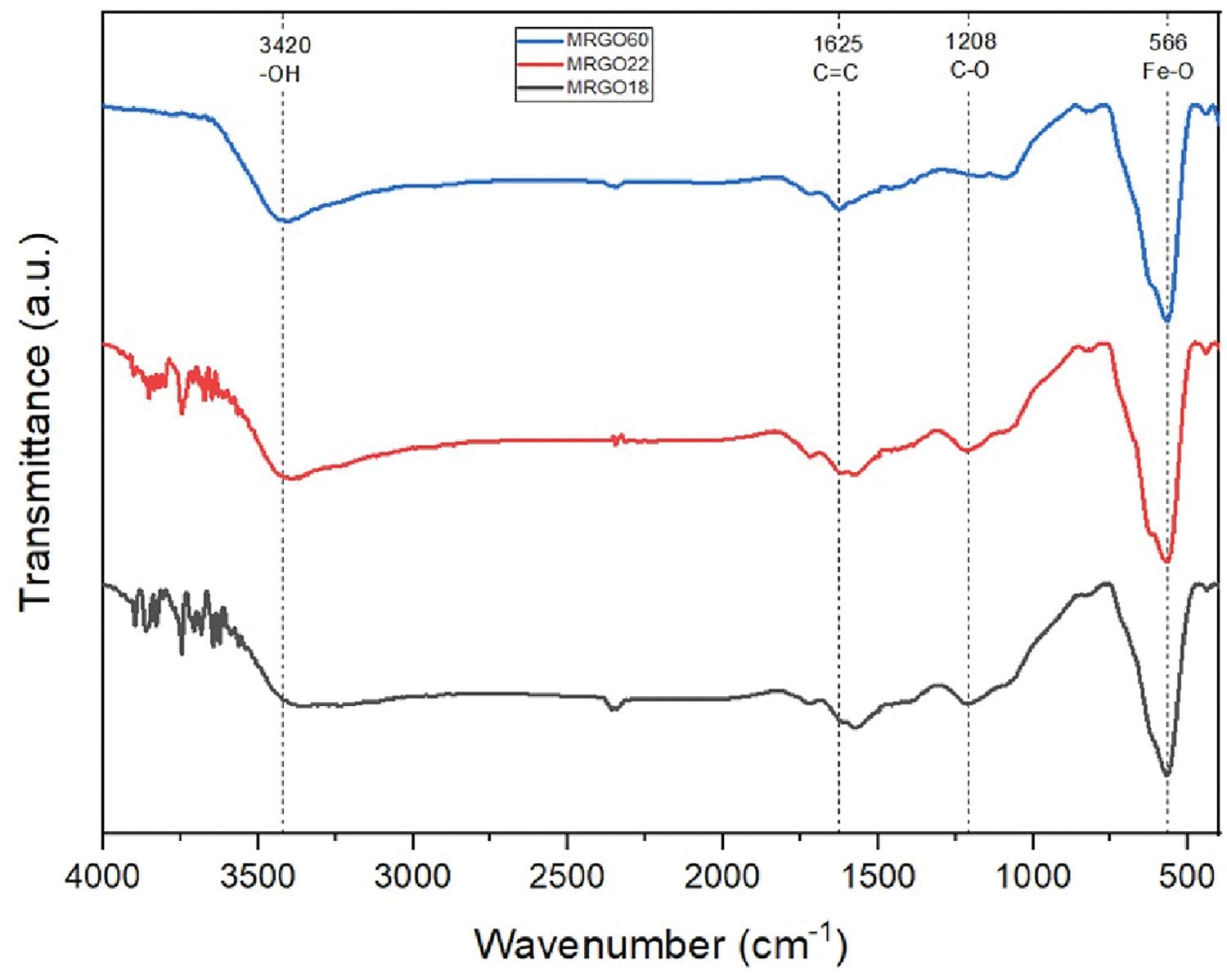
FTIR spectra for M-rGO60, M-rGO22, and M-rGO18.

FTIR spectroscopy analysis was conducted to further understand the composition of the samples from their chemical bonding and structure. It also assisted in identifying the formation of the magnetite nanoparticles onto the rGO sheets. This analysis further consolidated and solidified the success in the formation of the M-rGO nanoparticles. Referring to Fig 3, the M-rGO samples show four characteristic peaks that highlighted the chemical bonding within the sample which were the hydroxyl (-OH), aromatic C = C stretching, epoxy (C-O), and Fe-O at approximately 1208, 3420, 1573 and 566 cm^-1^, respectively. All three samples show the characteristic peak at 566 cm^-1^ which gives evidence that magnetite nanoparticles were able to be successfully stacked on the surface of the rGO nanosheets. The FTIR results are similar for the three M-rGO samples produced during in-situ chemical synthesis [13, 14]. When comparing the Fe-O peaks, it was detected that the absorption peaks for the samples increased as the amount of magnetite within the sample increased. At a ratio of 1:60, the absorption peak at Fe-O for M-rGO60 was at 40.51% while M-rGO22 and M-rGO18 were only at 39.4 and 33.63%, respectively.

Another indication that GO was successfully reduced to rGO is the absence of any GO characteristic peaks within the FTIR analysis such as carboxylic acids (O = C-OH) and anhydride groups (CO-O-CO) at 1732 cm^-1^ and 1074 cm^-1^, respectively. The process was able to reduce the amount of oxygen-functionalized groups as an effort to further minimize the number of these groups to mimic pure graphene’s characteristics [17]. Besides these oxygen groups, the removal of sp^3^ carbons in the carboxylic acid was also able to generate similar traits to graphene [17].

The removal of these oxygen-functionalized groups can further increase the overall conductivity of the carbon-based surface in M-rGO as the contact and charge transfer resistances will be reduced [10]. Following the removal of oxygen functionalized groups [17], the hydroxy and carbonyl groups can provide a higher capacitance compared to carboxylic acid and anhydride groups [10]. With the reduction of GO into rGO, the FTIR analysis pointed out that only the stretching vibration of hydroxyl (-OH) and epoxy (C-O) oxygen functionalized groups remained while anhydride and carboxylic acid groups from GO were undetectable. This indicates the successful reduction undergone by GO into rGO. The removal of these groups will decrease the contact resistance that occurs between the electrode surface and the current collector. In addition, the removal of these unwanted oxygen-functionalized groups can improve the charge transfer mechanism that occurs via the ionic channels during the formation of EDLs [18]. In contrast, certain oxygen-functionalized groups are to remain such as hydroxyl as they can improve the wettability of the M-rGO samples. This allows for improved ion diffusion rates when M-rGO-based electrodes are immersed in aqueous electrolytes [10].

#### 3.1.4 Characteristic peaks of D- and G-band of M-rGO nanocomposites using Raman spectroscopy

**Fig 4:**
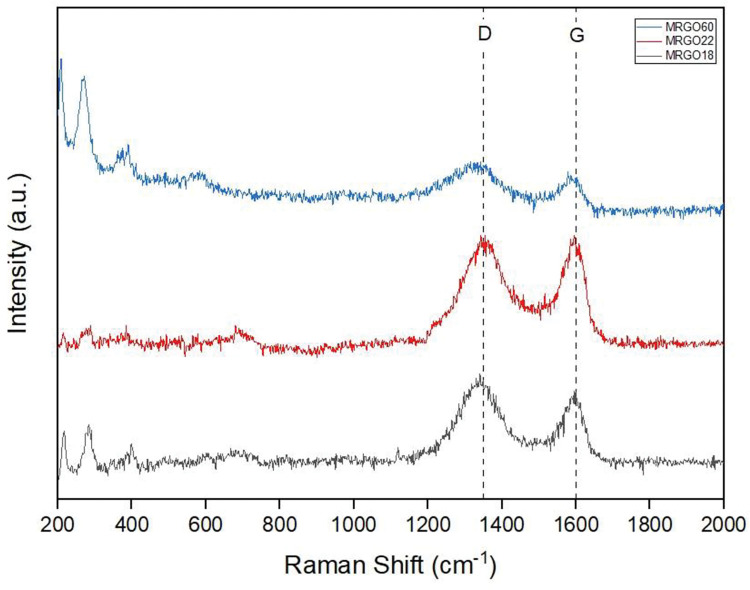
Raman spectra of M-rGO60, M-rGO22, and M-rGO18.

Raman spectroscopy was utilized as a method of further observation of the formation of magnetite nanoparticles onto the rGO nanosheets. This analysis method is useful as it is very sensitive to any changes that occur within a carbon-based material’s structure. Referring to Fig 4 compares the Raman spectra of M-rGO18, M-rGO22, and M-rGO60. The spectra demonstrate two characteristic peaks, more commonly known as the D band and the G band. The G band represents the spectra of the graphene material that originate from the sp^2^ carbon atoms while the D band represents the structural defects that occur within the material due to the disordered sp^3^ carbons [19].

For all samples, the characteristic peaks of the G band are observed at approximately 1590 cm^-1^. Meanwhile, the D band for each respective sample was detected at different Raman shifts whereby M-rGO18 shows its D band at 1340 cm ^-1^, M-rGO22 at 1356 cm ^-1,^ and M-rGO60 at 1319 cm ^-1^. This shift in the D band can occur during the functionalization and reduction of the oxygen-functionalized groups during the reduction process [20]. Thus, a broader D band could indicate that the samples were able to reduce a higher amount of oxygen-functionalized groups as the difference in the intensities at these shifts highlighted the degree of reduction during the process. Besides that, the broadening of the D band was detected most obviously in M-rGO60. This broadening indicated that defects were more clustered in specific regions instead of being distributed across the GO nanosheet causing a less intense D band [20]. In addition to the characteristic peaks displayed by the rGO, the presence of the four characteristic peaks originating from Fe_3_O_4_ located approximately at 281 cm^-1^, 284 cm^-1^, 400 cm^-1^, and 700 cm^-1^ also confirmed the functionalization of magnetite onto the GO nanosheet.

Raman spectroscopy can demonstrate the intensity ratio of the D and G peaks of the samples. The I_D/_I_G_ illustrates the degree of disorder that occurs within the graphene layers and the density of defects in the GO nanosheets. Another indication of the successful attachment of the magnetite nanoparticles onto the nanosheets is the D band possessing a higher intensity compared to the G band at its respective Raman shifts. The I_D/_I_G_ for M-rGO18, M-rGO22, and M-rGO60 were 1.36, 0.88, and 1.14 respectively. This indicated that M-rGO22 was able to produce a more ordered crystalline structure with minimal structural defects. While a lower defect density would be able to produce improved electrical conductivity properties with a larger surface area [20] but due to M-rGO22s high C/O ratio, caused the sample to have a smaller surface area than M-rGO18. In contrast, while the C/O ratio for M-rGO18 and M-rGO60 in the EDS analysis were comparable to each other with a small percentage of difference, the average particle size of M-rGO60 is much bigger causing it to have the smallest surface area among the three samples.

#### 3.1.5 Pore volume, diameter and surface area of M-rGO nanocomposites using N2 adsorption-desorption analysis

N_2_ adsorption-desorption analysis was conducted to study the pore volume, pore diameter, and surface area of the samples. The surface area plays a vital role in the electrochemical properties of the M-rGO samples as each parameter can further alter the energy storage performance. The surface area of the sample can be correlated with many factors such as the size of the nanoparticle, the C/O ratio, the I_D/_I_G_ ratio, and the intensity of the oxygen-functionalized groups remaining on the surface of the sample. The amount and intensity of oxygen functionalized groups influence the surface area of the M-rGO samples [20]. Referring to the previous FTIR analysis, it was observed that M-rGO60 had the highest intensity of the remaining oxygen-functionalized groups compared to other samples. M-rGO60 had an intensity of 46.64% and 49.42%, respectively, for its hydroxyl and epoxy group in comparison to M-rGO18 (38.2% and 37.94%) and M-rGO22 (44.57% and 46.17%). Hence, this resulted in M-rGO60 having a smaller surface area. Despite its high intensity, the remaining oxygen-functionalized groups such as hydroxyl (-OH) have been said to improve the overall electrode capacitive performance by improving the wettability allowing for ion migration and access. In addition, the I_D/_I_G_ was also a significant parameter, where a smaller defect density increases the overall surface area. Following the calculated I_D/_I_G_ values and C/O ratios, M-rGO60 would have the smallest surface area with the best capacitive performance among the three samples.

Referring to Table 2, the surface area, pore volume, and pore size can be seen for each of the samples. The decrease in pore volume across the three samples could be attributed to the increase in magnetite in each sample. This could indicate that most of the pores on the GO nanosheet would be occupied with magnetic nanoparticles [18]. The author also stated that a better dispersion of the magnetic nanoparticles on top of the GO nanosheet can also produce a larger surface area. This is evident in previous FESEM pictures as M-rGO18 magnetite nanoparticles were more evenly well distributed with less packing and agglomeration among the neighbouring particles.

**Table 2:** Surface area, pore volume, and pore diameter of M-rGO18, M-rGO22, and M-rGO60.

Sample Parameter	M-rGO18	M-rGO22	M-rGO60
Surface area (m^2^/g)	125.9758	98.3111	59.6019
Pore volume (cm^3^/g)	0.007402	0.006248	0.0018641
Pore diameter (nm)	8.9115	10.2107	17.1454

Besides that, the results align with previous FESEM results whereby the average particle size for each of these samples increased from M-rGO18 to M-rGO60. As the size of the average particle increases, the overall surface area would decrease. However, when comparing the results of the N_2_ adsorption-desorption analysis, observed that M-rGO60 had the largest pore diameter.

#### 3.1.6 Magnetic behaviour of M-rGO nanocomposites using vibrating sample magnetometry

**Fig 5:**
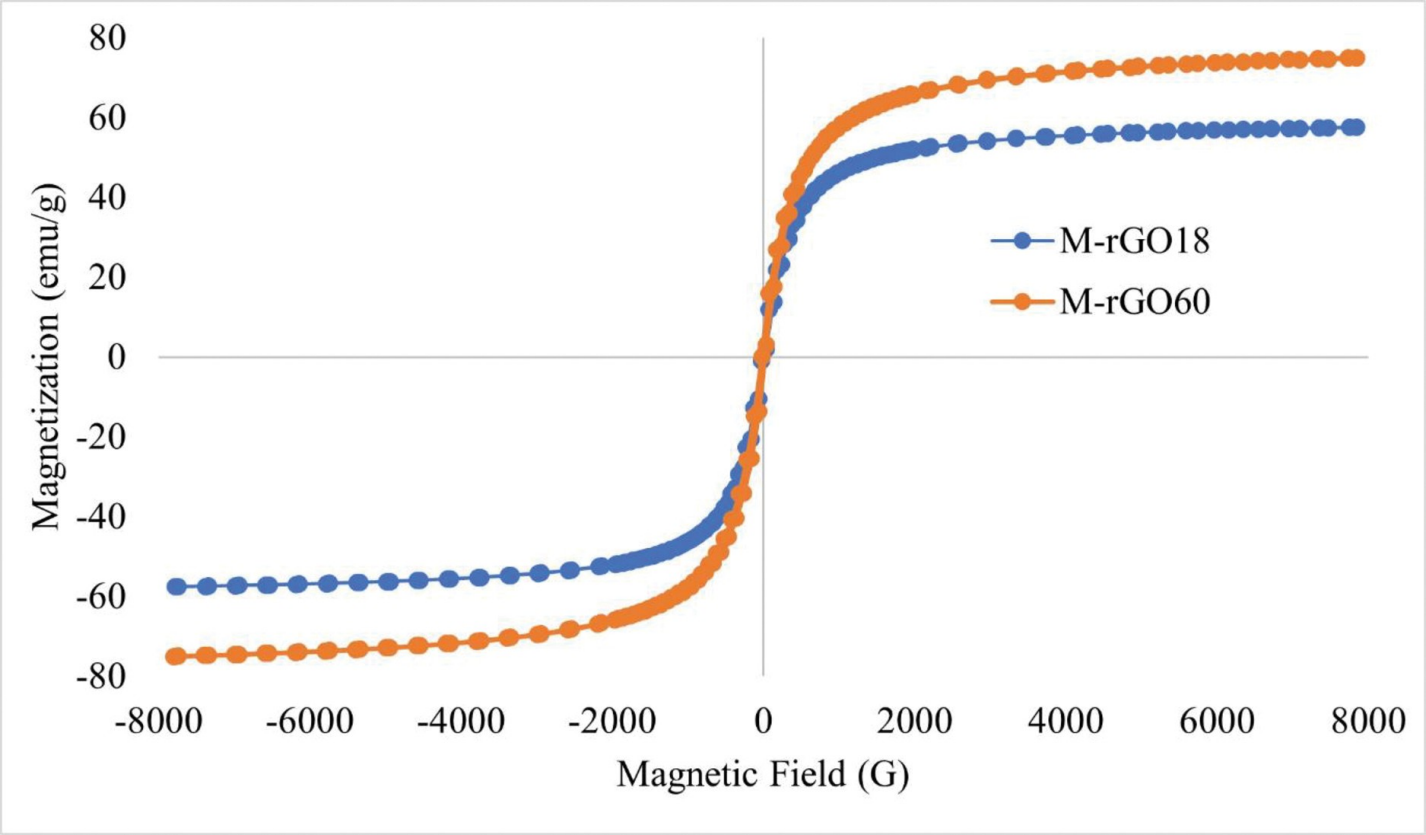
Magnetic curves of M-rGO18 and M-rGO60.

Vibrating sample magnetometry (VSM) was conducted to study the magnetic behavior of the prepared nanocomposites. For this analysis, M-rGO18 and M-rGO60 were selected to observe the difference in magnetic behavior. The magnetization (Ms) of M-rGO18 and M-rGO60 are 57.59 and 75.00 emu/g, respectively. The magnetic curves are shown in Fig 5, where both samples were able to exhibit a typical sigmoid-like or S-like curve [7].

The magnetite coercivity of M-rGO18 and M-rGO60 were 18.068 and 20.594 G. The magnetic coercivity represents the measure of the resistance of a material to resist the influence of an external magnetic field without losing the nanocomposites’ magnetism. Thus, the increase in the weight ratio of magnetite within the sample leads to an increase in the magnetic coercivity causing an increase in the required magnetic field strength needed to demagnetize the M-rGO nanocomposites. On the other hand, the magnetic retentivity of M-rGO18 and M-rGO60 were 2.3023 and 3.3447 emu/g, respectively. This indicates that the higher weight ratio of magnetite in M-rGO60 compared to M-rGO18 allowed the sample to retain its magnetism even in the absence of the external magnetic field. The coercivity and retentivity were able to highlight the superparamagnetic nature of the nanocomposites [7]. It is observed that a larger weight ratio of magnetite would further increase the sensitivity of the sample towards an external magnetic field as seen in the hysteresis loops in Fig 5.

### 3.2 Electrochemical measurement

#### 3.2.1 Cyclic voltammetry

**Fig 6:**
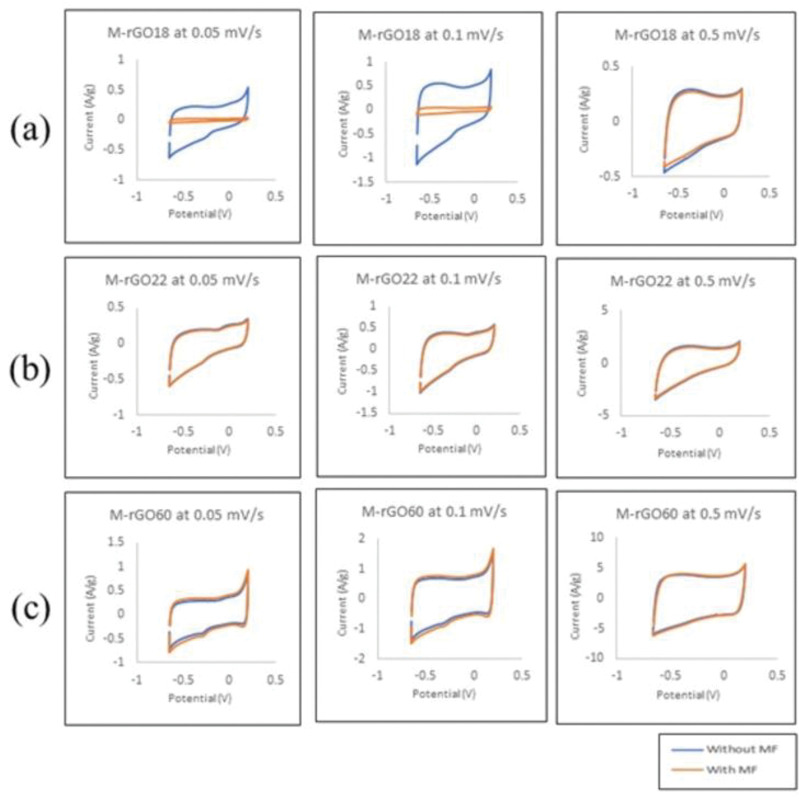
Cyclic voltammetry curves without (blue line) and with (orange line) magnetic field at 0.05, 0.1, and 0.5 V/s for (a) M-rGO18, (b) M-rGO22 and (c) M-rGO60.

Cyclic voltammetry for all samples was conducted at different scan rates with and without an external magnetic field as shown in Fig 6. To further observe the change in capacitance of each M-rGO sample under a magnetic field, the specific capacitance at each scan rate was calculated with Eq 2:

$$C_{s}=\frac{1}{sm(V_{a}-V_{b})}\int_{V_{b}}^{V_{a}}idV$$ (2)

Where s is the scan rate, m is the mass of the active material onto the electrode, i is the current density, and V_a_ and V_b_ represent the integration limits of the voltametric curves produced.

Based on the CV curves, the black and red line differentiates the CV curves without and with the influence of an external magnetic field. Previous literature predicted that CV curves under a magnetic field would be much more comprehensive compared to regular CV curves as the magnetic field would further enhance electron transportation efficiency, thus increasing overall capacitance. Despite this initial prediction, it was observed that the CV curves for M-rGO18 demonstrated the opposite of this statement. Meanwhile, the two CV curves for M-rGO22 have very similar areas under the curve. M-rGO60 was the only sample with the most noticeable difference when the graphs at each scan rate were compared.

For the case of M-rGO18, the stagnant nature of its capacitance could be justified due to the saturation of the magnetic field. As M-rGO18 has the smallest content of magnetite, the magnetic field could have possibly been too strong. Higher magnetic fields could induce electron deflection towards the edge of the electrode due to the large drift velocity experienced by the electron caused by the applied magnetic field. Hence, charge accumulation at the specific area of the electrode would result in a saturated value of the capacitance [21]. M-rGO18 electrode was able to have better capacitive performance without the presence of the magnetic field as there was no accumulation occurring that could disrupt the formation of the EDLs. For example, the calculated specific capacitance without and with the magnetic field at 0.1 V/s was 10.23 and 9.71 F/g, respectively, with a 5.08% reduction.

Subsequently, the CV curves obtained by M-rGO22 with the magnetic field are very similar to those without the magnetic field. As M-rGO22 contained more magnetite than M-rGO18, it would not experience the magnetic saturation as evident as M-rGO18, thus, the saturation capacitance versus its normal capacitance had a smaller percentage of difference. For example, the calculated specific capacitance at 0.05 V/s was 8.94 and 8.61 F/g with only a 3.4% reduction. Henceforth, the content of magnetite in M-rGO22 could not have displayed the positive effect of the additional magnetic field as visible. Consequently, this could have indicated that the amount of magnetite versus the strength of the magnetic field is an important factor to consider when applying a magnetic field with M-rGO nanocomposites.

For M-rGO60, the CV curves display a more evident difference when the electrode was placed under the influence of the magnetic field. A higher weight ratio of the magnetite within the nanocomposite resulted in improved capacitive performance under the magnetic field. As a result, the calculated specific capacitance without and with the magnetic field at 0.1 V/s was 13.29 F/g and 15.64 F/g, increasing the capacitance by 18%. The presence of an external magnetic field allows for the improvement of electron transportation at the electrolyte/electrode interface [22]. Due to the Lorentz force generated by the magnetic field, this further allowed the movement of ions into the electrode surface. Not only does this lead to an increase in capacitance, but this also reduces the overall charge transfer resistance experience by the electrode. Despite no noticeable increase in the capacitance for other samples, resistance is still expected to be decreased even at saturation.

Additional calculated specific capacitances of all samples at other scan rates are listed in Table 3. The maximum specific capacitance obtained for M-rGO60 at 0.1 V/s is 16.36 F/g under magnetic field influence. A further observation from the calculated specific capacitance includes that the specific capacitance is lowest at the highest scan rate of 0.5 V/s. This phenomenon was contributed due to the limitation of effective sites available for electrolyte ion penetration when at high scan rates.

**Table 3:** Calculated specific capacitance for all samples at different scan rates.

Sample	Scan rate (V/s)	Specific Capacitance (F/g)	
		(Without Magnetic Field)	(With Magnetic Field)
M-rGO18	0.05	9.89	9.50
	0.1	10.23	9.71
	0.5	9.98	8.59
M-rGO22	0.05	8.94	8.61
	0.1	8.03	7.68
	0.5	5.61	5.28
M-rGO60	0.05	13.29	15.64
	0.1	14.41	16.36
	0.5	14.51	15.24

#### 3.2.2 Galvanostatic charge-discharge

**Fig 7:**
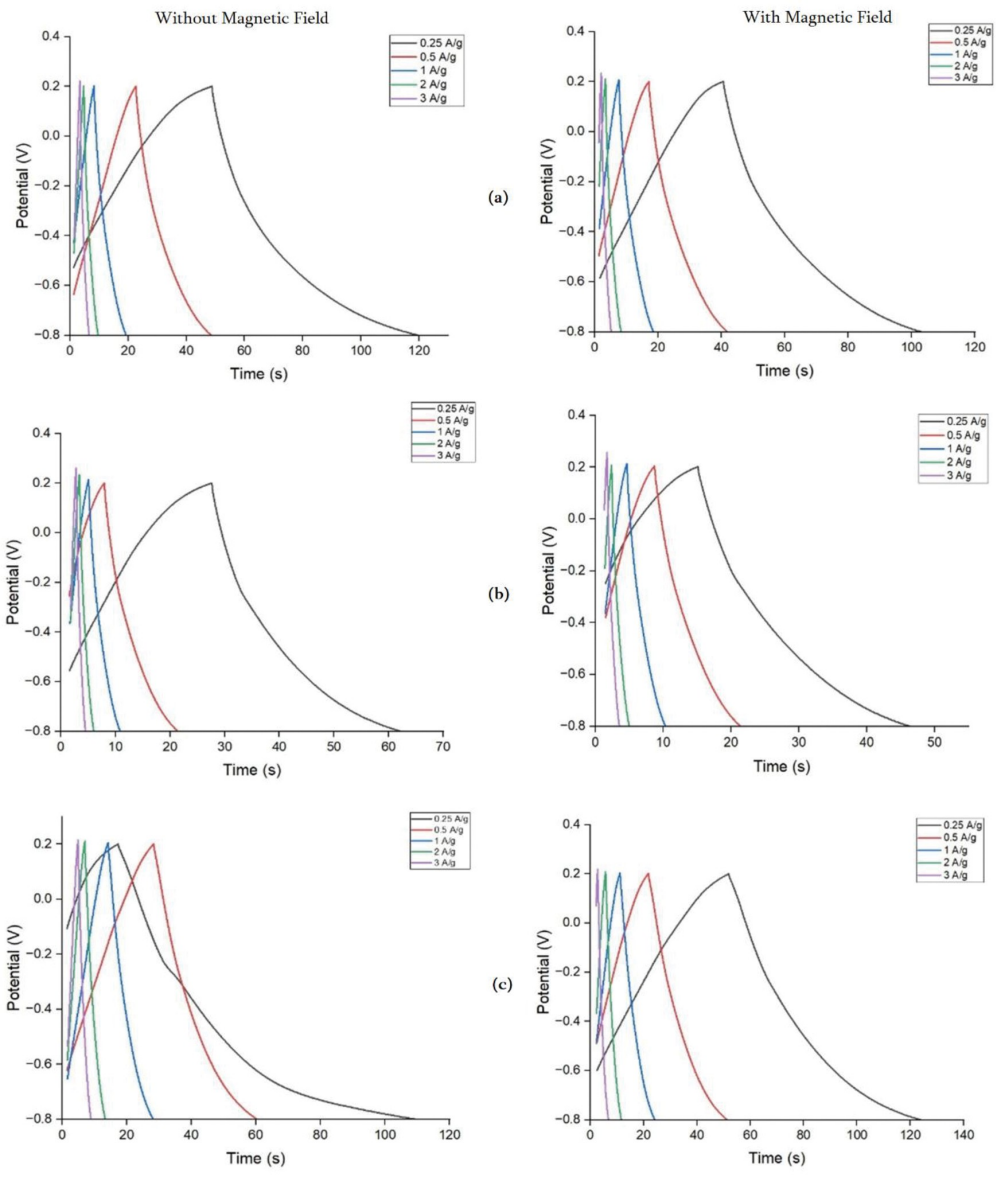
Galvanostatic charge-discharge curves without and with the magnetic field of a) M-rGO18, b) M-rGO22, and c) M-rGO60 at various current densities in 1 M KOH solution.

The galvanostatic charge-discharge (GCD) curves of all M-rGO samples without and with the magnetic field are observed in Fig 7. This method was utilized to further evaluate the specific capacitance of these M-rGO samples at current densities of 0.25, 0.5, 1, 2 and 3 A/g.

The charge-discharge curves for all the samples produced a symmetrical triangle-shaped curve which is a good indication of the capacitive behavior of these M-rGO nanocomposites. M-rGO18 and M-rGO22 electrodes under the influence of the magnetic field were unable to increase the duration of the discharging and it reduced the capacitive performance into a saturated state despite the previous resistance reduction. Interestingly, the discharge times for M-rGO18 were longer than the duration for M-rGO22 as observed in the GCD curves presented by both samples. M-rGO18 discharge time was 71.4 s at 0.25 A/g without a magnetic field, while M-rGO22 was 62.41 s. However, this aligns with the previous data obtained from the EDS and surface area analysis, whereby M-rGO22 contained the highest C/O ratio and smaller surface area, thus resulting in poorer capacitive performance. This similar conclusion was reached for other electrochemical testing as it has been verified that the C/O ratio and surface area play a major role in the capacitive properties of the electrode material. Contrary to the results experienced by M-rGO18 and M-rGO22, the discharging time of M-rGO60 at 0.25 A/g increased from 109.85 s to 124.07 s when the magnetic field was introduced.

The specific capacitance was calculated according to discharge curves at different current densities using Eq 3 and listed in Table 4:

$$C_{s}=i\frac{\Delta t}{m\Delta V}$$ (3)

Where i is the discharge current, Δ*t* is the discharge duration, and *ΔV* is the potential window.

**Table 4:** Specific capacitance at different densities for M-rGO18, M-rGO22, and M-rGO60.

Sample	Current density (A/g)	Specific capacitance (F/g)	
		(Without magnetic field)	(With magnetic field)
M-rGO18	0.25	21.00	18.47
	0.5	15.29	14.59
	1	13.18	12.94
	2	11.76	11.77
	3	11.29	11.29
M-rGO22	0.25	10.24	9.24
	0.5	8.00	7.53
	1	7.06	6.83
	2	6.59	6.12
	3	6.35	7.06
M-rGO60	0.25	21.24	27.24
	0.5	17.42	18.94
	1	15.53	16.71
	2	14.12	15.06
	3	14.82	14.12

Based on the calculated specific capacitance results, the specific capacitance decreased as the current density increased. The specific capacitance decreased due to the redox reaction’s overall kinetics moving moderately to keep pace with the fast potential [22]. Based on initial observations, the specific capacitance of M-rGO60 is improved mostly under the influence of a magnetic field. The specific capacitance for M-rGO60 under a magnetic field increased by 28% from 21.24 to 27.24 F/g. Due to the saturation of the magnetic field onto M-rGO18 and M-rGO22 electrodes, the specific capacitance was unable to increase with the magnetic field. On the other hand, M-rGO60 demonstrates the positive effects of M-rGO under a magnetic field. In line with the characterization results, M-rGO60 was expected to have better capacitive performance due to its larger pore size, lower C/O ratio, and higher intensity of hydroxyl functional groups as these parameters allowed larger channels or pores for electrolyte ion diffusion and migration. This further enhanced the effective contact between the electrode and the electrolyte, thus reducing the overall contact resistance between the current collectors.

#### 3.2.3 Electrochemical impedance spectroscopy

**Fig 8:**
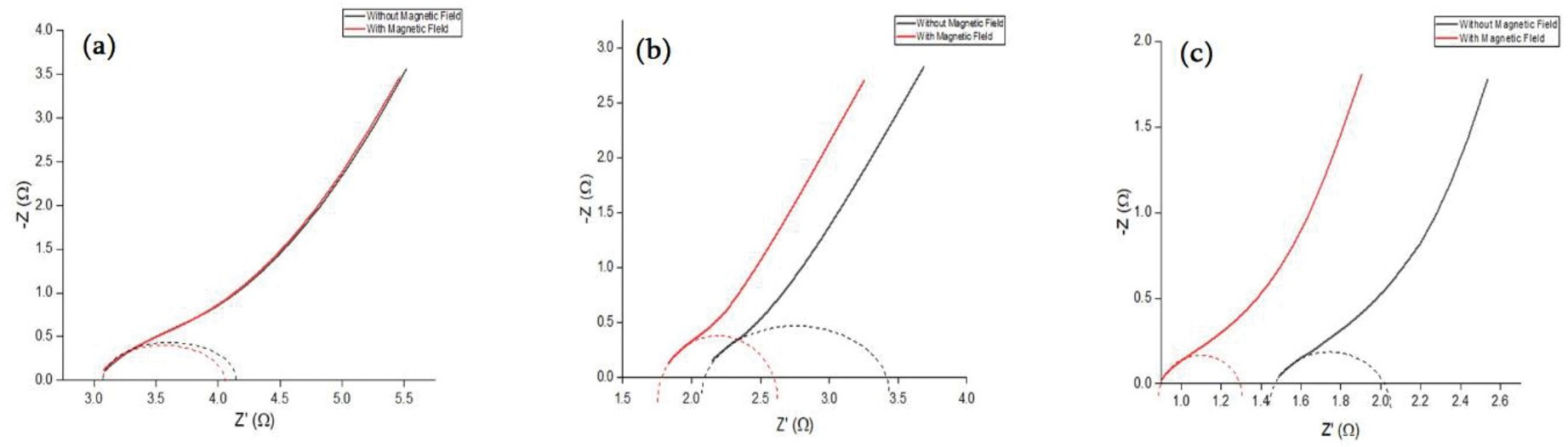
Nyquist plots of a) M-rGO18, b) M-rGO22, and c) M-rGO60.

EIS was utilized to study the electron transport and diffusion characteristics of the M-rGO electrodes. As shown in Fig 8, the Nyquist plots of each sample can be observed without and with the magnetic field. The Nyquist plots illustrated the three different regions under different frequencies with low, medium, and high-frequency regions [23]. The semicircle high-frequency regions demonstrated the contact resistance between the electrode and current collector and the charge transfer impedance at the electrode/electrolyte surface. The low-frequency region where the EIS shows a vertical line that represents the capacitive behavior of the overall capacitor.

To observe the resistance reduction resulting from the addition of an external magnetic field, the diameter of the semicircle representing the charge transfer resistance (R_ct_) was obtained. The corresponding R_ct_ is listed in Table 5.

**Table 5:** Charge transfer resistance without and with applying an external magnetic field for all samples.

Sample	R_ct_ (Ω)		Resistance reduction
	(Without magnetic field)	(With magnetic field)	
M-rGO18	1.20	1.16	0.04
M-rGO22	1.30	0.78	0.52
M-rGO60	0.6	0.4	0.2

From the table, the results highlighted that the largest value of the charge transfer impedance is 1.30 Ω for the M-rGO22 nanocomposite. In line with the previous EDS analysis, results have shown that M-rGO22 contained the highest C/O ratio. This ties well with the understanding that an increase in the C/O ratio within the sample will result in a higher overall resistance within the nanocomposite. When comparing the C/O ratio obtained by M-rGO18 and M-rGO60, both samples demonstrated the same pattern, whereby M-rGO60 had the smallest charge transfer impedance of only 0.6 Ω among the three composites. The straight line at lower frequencies for M-rGO60 was more vertical than its counterparts once the magnetic field was applied, indicating improved capacitive performance.

Notably, the capacitive behavior of the material should improve as the R_ct_ decreases as the magnetic field is applied. However, when comparing these results, M-rGO22 had the worst capacitive performance despite having the largest reduction in its R_ct._ These findings can be following the problem of saturation experienced by both M-rGO18 and M-rGO22 due to the lower amount of magnetite within the sample. Similarly, M-rGO18 could only experience a minimal reduction (about 3.3%) of the charge transfer resistance. Alternatively, this could indicate that while the amount of magnetite in M-rGO22 was sufficient to be influenced by the magnetic field compared to M-rGO18, the overall strength of the field applied was too high, resulting in a considerable reduction of resistance with no increasing capacitance as it remained saturated.

Overall, the electrochemical impedance spectroscopy results clearly illustrate that the charge transfer impedance, which takes place at the electrode-electrolyte interface, is notably reduced when the electrode is subjected to a magnetic field. Specifically, significant reductions in charge transfer impedance were observed for samples like M-rGO22 and M-rGO60, with reductions of 0.52 Ω (40%) and 0.2 Ω (33%), respectively. These findings strongly suggest improved ion transport during the electric double layer (EDL) formation process. This reduction in impedance also corresponds to enhanced diffusion and migration of electrolyte ions into the porous electrode structures, thereby facilitating charge storage.

## 4. Conclusion

In conclusion, the successful synthesis and characterization of magnetite-reduced graphene oxide (M-rGO) nanocomposites revealed important parameters influencing their capacitive performance. Factors such as the carbon-to-oxygen (C/O) ratio, the ratio of D band intensity to G band intensity (ID/IG), surface area, and particle size were identified as contributing factors. M-rGO60 demonstrated significant improvement in electrochemical performance under influence of the magnetic field, leading to a roughly 28% increase in specific capacitance. While M-rGO18 and M-rGO22 reached saturation under the strong magnetic field, all three samples exhibited reduced resistance. Thus, optimizing magnetite content and induced magnetic field strength is crucial for maximizing capacitance. A magnetic field is able to reduce the ion transfer resistance, improve ion migration and increase overall capacitance. However, other influencing factors must also be considered. Overall, integrating a magnetic field in M-rGO-based supercapacitors hold promise for better performance.
