# Correction: Enhancing capacitive performance of magnetite-reduced graphene oxide nanocomposites through magnetic field-assisted ion migration

**DOI:** 10.1371/journal.pone.0330901

**Published:** 2025-08-22

**Authors:** Nur Alya Syakirah Abdul Jalil, Eslam Aboelazm, Cheng Seong Khe, Gomaa A. M. Ali, Kwok Feng Chong, Chin Wei Lai, Kok Yeow You

The Funding statement for this article is incorrect. The correct Funding statement is as follows: This research is supported financially by the Ministry of Higher Education (MOHE) under the Fundamental Research Grant Scheme (FRGS/1/2023/STG05/UTP/02/1). The funders had no role in study design, data collection and analysis, decision to publish, or preparation of the manuscript.
